# Technical and Socioeconomic Potential of Biogas from Cassava Waste in Ghana

**DOI:** 10.1155/2015/828576

**Published:** 2015-11-18

**Authors:** Francis Kemausuor, Ahmad Addo, Lawrence Darkwah

**Affiliations:** ^1^Department of Agricultural Engineering, Kwame Nkrumah University of Science and Technology (KNUST), Kumasi, Ghana; ^2^The Energy Center, KNUST, Kumasi, Ghana; ^3^Department of Chemical Engineering, KNUST, Kumasi, Ghana

## Abstract

This study analyses technical potential and ex ante socioeconomic impacts of biogas production using cassava waste from agroprocessing plants. An analysis was performed for two biodigesters in two cassava processing communities in Ghana. The results showed that the two communities generate an excess of 4,500 tonnes of cassava peels per year. Using approximately 5% of the peels generated and livestock manure as inoculum can generate approximately 75,000 m^3^ of gas with an estimated 60% methane content from two separate plants of capacities 500 m^3^ and 300 m^3^ in the two communities. If used internally as process fuel, the potential gas available could replace over 300 tonnes of firewood per year for cassava processing. The displacement of firewood with gas could have environmental, economic, and social benefits in creating sustainable development. With a 10 percent discount rate, an assumed 20-year biodigester will have a Net Present Value of approximately US$ 148,000, 7-year Payback Period, and an Internal Rate of Return of 18.7%. The project will create 10 full-time unskilled labour positions during the investment year and 4 positions during operation years.

## 1. Introduction

Cassava is a major food crop, especially in Africa. About 60% of the cassava produced all over the world is used for human consumption [1] and is consumed in different forms. The second largest consumer of cassava is the animal food industry, which uses about 33% of the world production. The remaining 7% is used by industries such as textile, paper, and fermentation [1]. In 2012, cassava production amounted to more than 260 million tonnes globally [2]. Nigeria is the largest producer, contributing over 20% to global production. Ghana is the sixth largest producer with 5.5% of production, amounting to about 14.5 million tonnes in 2012. In terms of production per capita, cassava is the highest cultivated crop in Ghana with per-capita production of 0.6 tonnes, compared to 0.3 tonnes per capita in Nigeria.

In Ghana, cassava is one of the critical staple foods and is processed into/used to prepare several foods, many of which can be stored for up to several months. The more common foods made from cassava are* fufu*,* agbeli kaklo*,* gari*, and* kokonte*.* Fufu* is a staple food in most households in the southern parts of Ghana and is considered a delicacy that is also served in most restaurants across the country. Among the industrial uses, starch is currently the more common outputs. Efforts to use cassava to produce ethanol are also ongoing [3]. Among the food uses,* gari* is produced on commercial basis, for both local consumption and export. Processing cassava into* gari *is an agroindustrial activity that takes place on a small- to medium-scale basis. Small-scale processing, often up to a few tonnes of cassava per year, is done at the household level. Medium-scale production is done in agroprocessing plants that process up to a few thousand tonnes per year. It is estimated that about 25% of cassava harvested in Ghana is processed into* gari *[4] in communities in the southern parts of the country.

In order to maximise output of cassava and other root and tuber crops in Ghana, the “Root and Tuber Improvement and Marketing Programme” (RTIMP) (the programme is a follow-up of the Root and Tuber Improvement Programme (RTIP) which was implemented from 1999 to 2005. RTIMP is being sponsored for a period of 8 years (2007–2014) and was expected to be implemented across 60 districts but this has now been scaled up to 90 districts) was instituted in 2007. RTIMP is supported by the International Fund for Agricultural Development (IFAD) and the Government of Ghana (GoG) through the Ministry of Food and Agriculture (MOFA) and seeks to develop downstream activities like processing and marketing of cassava. The programme has assisted local entrepreneurs to establish cassava processing factories (processing cassava into* gari*) in communities where cassava is produced on relatively larger scale. The factories that receive assistance are known as “Good Practice Centres (GPC),” because they follow a strict code of practice to ensure the production of hygienic* gari* that can also be exported to neighbouring countries.

In most cassava processing communities, several tonnes of cassava peels are generated as a waste product from the processing activity. With an expected increase in cassava production, it is also expected that waste generation will also continue to rise. Even though cassava peels can be used as feed for livestock, the quantities generated and the remoteness of many of the communities where processing takes place leave behind a lot of waste, which is left to rot or is burnt, with environmental consequences.

There is therefore the need to explore other measures to manage the waste accruing from the process in order to ensure good environmental management practices within the processing communities. To this end, RTIMP launched a project dubbed ProVACCA (PROmoting a Value Chain Approach to Climate Change Adaptation in agriculture in Ghana) in 2013 which has two principal aims (details of the project,* PROmoting a Value Chain Approach to Climate Change Adaptation in Agriculture in Ghana (ProVACCA)*, can be found at http://operations.ifad.org/documents/654016/17060381-148f-4291-b04a-24511050954e). The first aim is to prepare cassava and other root/tuber farmers to adapt to changing climate and by so doing ensure food security. The second aim is to encourage the use of cassava waste as fuel for cassava processing. To achieve the second aim, RTIMP is in the process of constructing pilot biodigesters or gasification plants in two communities to generate energy. This study aims to further explore the impacts of using cassava wastes to produce biogas in these communities.

The use of cassava wastes as a biogas substrate has been experimented, either as a standalone raw material or in combination with livestock manure. Cuzin et al. [5] have conducted an experiment on the production of biogas using cassava peels. Other researchers have carried out experiments using cassava peels in combination with other feedstocks. Some of the feedstocks that have been used include poultry manure [6–8], cow dung [7–9], zebra droppings [10], pig dung [7, 8, 11–13], and cowpea [9].

Currently, communities processing cassava use firewood as the main heating source, as is the case with many rural community agroprocessing activities in Ghana. Government's policy objective is to ensure that agroindustries shift from the use of firewood to more environmentally friendly fuels such as biogas for heating. The country's Strategic National Energy Plan [14] has proposed an increase in renewable and modern biomass energy in the final energy supply to achieve at least 10% penetration by 2020. This is also corroborated by the Renewable Energy Law of Ghana which was promulgated in 2011 [15]. However, the extent to which residues from processing plants could serve as feedstock for energy has not been the subject of much research in Ghana. This study therefore examines the technical and socioeconomic potential of generating methane from cassava waste to replace firewood, which is increasingly becoming scarce. The specific objectives are to

1. examine the availability of cassava waste from cassava processing and its potential for methane production;
2. perform financial assessment of producing methane from cassava waste;
3. assess job creation potential and other social benefits of biogas production from cassava process waste.

## 2. Materials and Methods

### 2.1. Study Area

**Figure 1:**
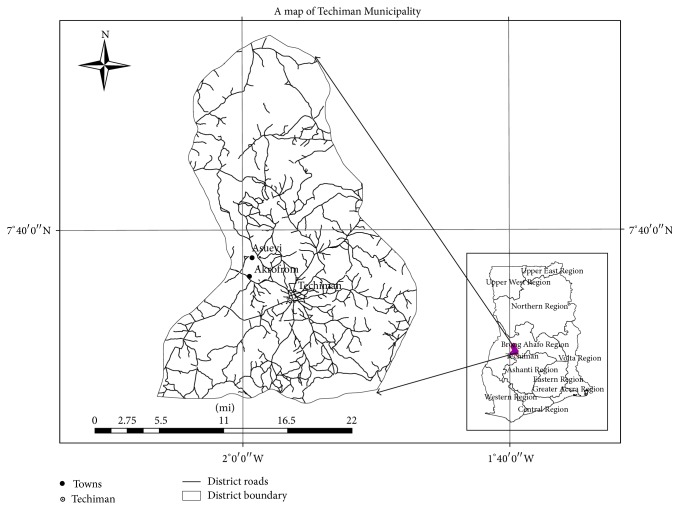
Map showing study locations.

The study was conducted in two agroindustrial processing sites in* Asueyi* and* Akrofrom*, both located in Techiman Municipality of the Brong Ahafo Region (see Figure 1). Techiman Municipality is a major cassava production district in Ghana. The two communities were selected for this study because they are also major cassava processing areas within Techiman Municipality. Both communities receive assistance from RTIMP and have been selected to benefit from a pilot bioenergy conversion plant. Both communities have similar socioeconomic characteristics. As of the last census in 2010,* Asueyi* community had a population of 2,402 and* Akrofrom* community had 1,505 people. Both communities are agrarian with the majority of residents engaged in farming activities. Farmers cultivate cassava, cocoa, and cashew, in addition to other staple crops and vegetables. Cassava is a major crop because of its commercial value as raw material for* gari *production. Cassava processing is a vibrant economic activity in both communities.

*Asueyi* community processes about 8,000 t of cassava per year, producing about 1,500 t of* gari*.* Akrofrom* community has two processing sites. However, data for this work was obtained from only one site, which processes an excess of 7,000 t of cassava per year. Between five and ten different cassava varieties are processed in both communities. Cassava is generally available all year round due to a planned cultivation and harvesting schedule. Occasional shortages may occur due to transportation or logistical challenges but not from shortage of the produce. Firewood is the only fuel for roasting and is purchased from suppliers. The study site in* Asueyi* had forty roasting points and* Akrofrom* community site had thirty-five. Each roasting point consists of a stove and roasting pan and is manned by one person.

### 2.2. Description of Cassava Processing Activity

**Figure 2:**
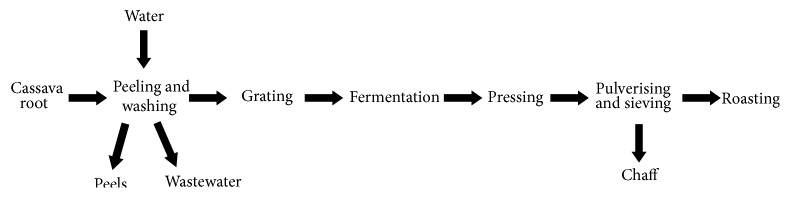
Flowchart for processing cassava into* gari*.

Figure 2 summarises the stages in cassava processing for* gari* production. The first stage is peeling and washing of the cassava root. The peeled cassava is then grated using a motorized cassava grater. The next stage is fermentation where the grated cassava is left to ferment for 24 hours at room temperature. The fermented paste is bagged and pressed to remove moisture using hydraulic screw presses. The coarse flour material is pulverized and then sieved to make it finer for roasting. The roasting is done manually in large, shallow stainless steel pans over a fire, with constant stirring. The stirring takes place for 20–30 minutes and is done with a piece of broken calabash or wooden paddle carefully designed for the purpose. The roasted* gari* is sieved to obtain granules of uniform size and bagged for marketing.

### 2.3. Assessment of Feedstock

The first stage in the analysis of energy potential from cassava waste is the assessment of quantities of waste generated. An experiment was performed to assess the availability of peels from each of the processing plants. The experiment was performed between April and June 2014. The assessment was performed for four varieties of cassava which were processed during the period of the study. For each variety of cassava, thirty randomly selected samples from three different truck deliveries (thus ten samples from each truck delivery to the plant) were weighed and peeled. The weight of the peels was then recorded. Peelers used in the experiment were randomly selected from among the existing peelers at the processing plants. As part of the assessment, observations were made of the existing uses of cassava peels during the study period to estimate amount of peels collected for feeding livestock and amount discarded. Peels from each of the cassava varieties were collected for moisture content determination. The moisture content (wet basis) was determined using the oven method [16].

A survey was conducted in the two communities to determine the availability of manure to serve as inoculum for biogas production. The survey was structured to solicit information on cattle housing systems and existing uses of manure. The questions ranged from numbers of cattle raised, housing conditions, existing uses of manure, and cost of manure.

### 2.4. Measurement of Firewood Use

In order to assess the amount of firewood used for* gari* processing, a fuel use experiment was conducted. Ten roasting points were purposively selected from each processing facility based on consent to participate and agreement to observe the rules of the experimentation. Fuel use experiment was performed from June 16 to 23 and June 25 to July 2, 2014, for* Asueyi* and* Akrofrom*, respectively. Experiment at each roasting point took seven full days, requiring daily visits for eight days. For each roasting point, an amount of firewood (in excess of the daily requirement) was weighed daily and the leftover at the end of the working day weighed again to determine how much was used. For each roasting point, the amount of* gari* roasted for the day was also weighed. The amount of firewood used and the corresponding* gari* roasted are used to determine the amount of firewood per a unit of* gari* roasted. Data was analysed and the mean of the firewood recorded.

### 2.5. Assessment of Biogas Production

Both thermochemical and biochemical technologies can be used to convert cassava waste into useful energy forms. The technologies available include anaerobic digestion, gasification, and pyrolysis. The products from each of these technological processes differ due to the production thermodynamic parameters. This changes the compositions of the various gases in each technology. Gasification process leads to the production of producer gas, which is composed primarily of carbon monoxide (CO), hydrogen (H_2_), and traces of methane (CH_4_). Pyrolysis leads to the production of char, bio-oil, and syngas, which is again a mixture of mainly CO and H_2_. Anaerobic digestion leads to the production of biogas, a gas composed principally of CH_4_ and carbon dioxide (CO_2_). Anaerobic digestion was considered for the production of biogas in this study because it is more matured and less complicated and there is local expertise for the construction and maintenance of anaerobic digesters.

Theoretical calculations on the composition of biogas produced were determined using the Buswell equation based on the chemical composition of the cassava peels. Data on the chemical composition of cassava peel was obtained from a recent laboratory compositional analysis of Ghanaian cassava peels for methane potential [17]. Before the substrate is introduced into a biodigester, the cassava peels will have to be reduced to particle size ≤1 mm [13]. High methane production efficiency can only be achieved with inoculum. Ensuring the right combination of cassava peels and animal manure is key to ensuring maximum yield of gas. Different combinations of cassava peel with manure from cattle, pigs, and poultry have been studied.

Adelekan and Bamgboye [8] found that mixing cassava peels with pig manure had better biogas yield than using either of these wastes as a standalone feedstock. Using 1 : 1 pig-manure-to-cassava-peel ratio had a gas yield three times higher than a ratio of 3 : 1. Ofoefule and Uzodinma [7] also investigated the effect of cattle, poultry, and pig manure on biogas yield of cassava peels. They found that mean gas yield increased from lowest 2.29 litres per total mass of slurry for cassava peels alone to highest 8.27 litres total mass of slurry when combined with pig manure. Adelekan and Bamgboye [8] experimented with different combinations of cassava peels and manure, using peels-to-manure ratios of 1 : 1, 2 : 1, 3 : 1, and 4 : 1. For all the manure types, the ratio 1 : 1 gave the highest yield of biogas, though the 2 : 1 ratio followed closely for all manure types. For cattle manure, for example, the 1 : 1 ratio yielded 21.3 L/kg-TS while the 2 : 1 ratio produced 19.5 L/kg-TS. Using the same weight of cassava peel alone produced paltry 0.6 L/kg-TS. Other studies, including Adelekan [18] and Oparaku et al. [11], have found similar results.

Due to the critical nature of manure requirement for effective gas production, a livestock production and housing survey was conducted in the two communities to determine manure availability. The survey considered livestock that were partially or fully housed where manure could be recovered for energy purposes. In the final analysis, a 2 : 1 cassava-peel-to-livestock-manure ratio was used for computation, in order to increase the efficiency of biogas production. While a 1 : 1 ratio appears to be the best combination based on experimental results presented above, the low level of manure production in the study communities informed the 2 : 1 ratio of peel to manure.

The methane potential (*P*_methane_) was estimated using (1) which is modified from Kemausuor et al. [19]:

(1) Pmethane=PARyBuswell.glu∗Cglu+yBuswell.hem∗Chem∗ηscale+∑i=1nPlive∗yman∗ηrec∗CTS∗yBMPi,

where *P*_AR_ is the amount of cassava peel available, *y*_Buswell_ is the methane potential calculated with Buswell's formula, *C*_glu_ is the concentration of glucan (cellulose or starch) in cassava peel, *C*_hem_ is the concentration of hemicellulose, *η*_scale_ is the average efficiency of continuous biogas production, *P*_live_ is the number of specific livestock population, *y*_man_ is manure produced of one specific livestock annually, *η*_rec_ is the recoverability of manure for specific livestock, *C*_TS_ is the total solids concentration of manure, and *y*_BMP_ is the methane potential of specific livestock manure. Factors *i* and *n* represent the manure and total number of manure types (based on the livestock type producing it), respectively, for which methane potentials are computed. The efficiency of biogas production is dependent on the inoculum, which in this case is livestock manure. The analysis assumes 60% recovery of manure from pigs and cattle, only during the period of housing. For example, cattle are housed only at night, so that 60% recovery of the manure produced during the night is considered. All of the manure produced during the day is not considered as being available since the cattle are then not housed.

### 2.6. Financial Feasibility Assessment

To determine the financial feasibility of the biodigester, Net Present Value (NPV), Internal Rate of Return (IRR), and Payback Period (PBP) were used as indicators. NPV is the sum of the present values of individual cash flows over the project lifetime. The IRR is the discount rate at which the incremental net benefit stream or incremental cash flow is equal to zero [20].

NPV is computed using

(2) $$\begin{array}{c} NPV=\overset{n}{\underset{t=1}{\sum }}\frac{B_{t}-C_{t}}{{\left(1+i\right)}^{t}}. \end{array}$$

IRR is computed using (3) and is the discount rate “*i*” such that

(3) $$\begin{array}{c} 0=\overset{n}{\underset{t=1}{\sum }}\frac{B_{t}-C_{t}}{{\left(1+i\right)}^{t}}, \end{array}$$

where *B*_*t*_ is the benefit in each year, *C*_*t*_ are the costs in each year, *i* is the interest (discount) rate, and *t* are numbers from 1,2, 3,…, *n*, where *n* is the number of years (life of biogas plant).

### 2.7. Social Benefit Analysis

One of the very important reasons for promoting the use of agroindustrial waste for energy production is to contribute to job creation and income generation for rural communities. These are some of the key indicators of success in bioenergy development [21]. The social benefit analysis assesses the number of jobs that could be created and the corresponding income from using agroindustrial waste to generate biogas.

## 3. Results and Discussion

This section presents the results from the study and discusses its implications for bioenergy development in Ghana.

### 3.1. Cassava Peel and Biogas Potential

The ratio of peels to cassava roots, based on the experiment conducted at the two processing plants, is shown in Table 1. The average peel-to-whole-cassava ratio obtained for four cassava varieties is 0.303 with a standard deviation of 0.016. This means that, for every tonne of cassava processed, approximately 300 kg of peels is obtained, ranging from 290 kg for* Esam* variety to 321 kg for* Dakwari* variety. The data obtained corroborates findings by the FAO [22] which states that about 250 to 300 kg of cassava peels is produced per tonne of fresh cassava root processed. However, the figure obtained is slightly higher than 0.25 peel-to-cassava-root ratio quoted by Jekayinfa and Scholz [23].

**Figure 3:**
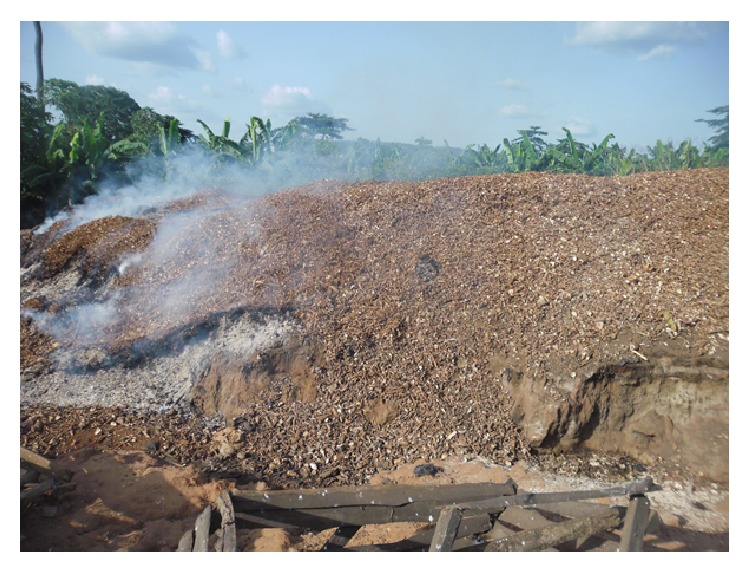
Pile of cassava peels undergoing open combustion.

Based on the peels-to-cassava-roots ratio shown in Table 1, peels generated in the two communities are shown in Table 2. Following the monitoring and interaction with the managers of the processing sites, it was estimated that about two-thirds of peels in* Akrofrom* are collected for livestock feeding and only one-third are collected in* Asueyi*. The lower collection rate in* Asueyi* can be attributed to the remoteness of* Asueyi* community with poor road connection. This makes it difficult and expensive for livestock farmers to regularly commute to the processing site for collection of peels, resulting in the creation of a huge pile of cassava peel within the community. The processing site has attempted to manage the waste by resorting to open combustion (see Figure 3) which has health implications for residents.

Based on the livestock survey, only 20 cattle and 20 pigs are kept in* Asueyi* community. In* Akrofrom* community, there are 45 cattle and 12 pigs. The cattle in both communities are housed only at night and allowed to open-graze during the day. The pigs are, however, housed 24 hours a day. The analysis for manure availability therefore estimated manure production from cattle for only half the day and a full day for pigs. Also, for the period when manure generation is considered, only 60% recoverability is estimated as it is not possible to collect all of the manure generated. Based on this analysis, only 46 t of manure is available from* Asueyi* and 75 t from* Akrofrom* community per annum.

The biogas production estimate is based on 2 : 1 peel-to-manure ratio following experiments conducted by Ofoefule and Uzodinma [7], Adelekan and Bamgboye [8], and Oparaku et al. [11]. Even though there are abundant cassava peels, the limited availability of livestock manure restricts the size of digester. Based on the 2 : 1 peel-to-manure ratio, only 4% of the peel generated in* Asueyi* and 7% from* Akrofrom* are estimated to be fed into a biodigester for biogas generation. This is very little, compared to an estimated 65% discarded cassava peels in* Asueyi *and 33% in* Akrofrom*. The combined feedstock (peels and manure) available in* Asueyi* can only support a 300 m^3^ plant whereas the feedstock in* Akrofrom* can support a 500 m^3^ plant. The annual potential of biogas from both communities is approximately 75,000 m^3^ of gas with an estimated 60% methane content. The ultimate aim for generating methane is to replace the use of firewood for* gari* processing. The potential for firewood replacement at the* gari* processing factories is shown in Table 2.

As mentioned earlier, it is estimated that a quarter of the cassava produced in Ghana is used for the production of* gari*. Meanwhile, all* gari* production factories rely on firewood which means that approximately 580,000 t of firewood was used for the production of roughly 682,000 t of* gari* in 2012 alone. The firewood used for* gari* production alone in 2012 amounts to approximately 13% of the estimated 4.56 million tonnes of firewood [24] consumed in Ghana in the same year. Exploring the use of cassava waste to produce fuel for the production of* gari* could replace firewood and result in social and environmental benefits.  Table 3 shows a projection of cassava production for Ghana with corresponding estimated amount that could be used for* gari* production.  Table 3 also shows the estimated firewood that could be used to process the potential* gari* using an average of the firewood amount used in the two communities. It is expected that close to 1.3 million tonnes of firewood could be needed for* gari* production by 2030 under a business-as-usual scenario. This figure is only indicative because there might be differences in other processing sites due to social practices, efficiency of roasting stoves, and other factors. However, this amount of firewood needed for* gari* processing by 2030 depicts the extent to which demand for firewood could rise in the* gari* production industry, with alarming consequences for the country's wood resources. Clearly, this could compete with rural households for scarce wood resources and calls for urgent attention.

### 3.2. Financial Assessment of Biogas Development

There are two options for using the methane gas: (1) internally for cassava processing and (2) by sale to households in the community to be used as cooking fuel. In large plants, both options could be pursued. The financial analysis is therefore performed from two perspectives. The first one investigates the extent to which gas produced could be used within the plant and its cost implications (compared to using firewood for roasting* gari*). The second analysis examines the profitability of generating the gas for sale to households within the community.

The capital cost for the biogas digester and other key financial indicators are summarised in Table 4. Capital cost for the 300 m^3^ plant in* Asueyi* is approximately US$ 91,000, rising to about US$ 151,000 for* Akrofrom*, where a 500 m^3^ plant is envisaged. The financial analysis is performed for a 25-year period, assumed to be the lifetime of the digester. The analysis from the fuel use experiment shows that it takes approximately 0.85 kg of wood to produce 1 kg of* gari*. Firewood is purchased at US$ 14.5 per tonne (using an exchange rate of 1 US$ to GHC 2.81 at the time fieldwork was conducted). Thus, at present value, it takes approximately US$ 12.325 of firewood to produce a tonne of* gari*. Taking* Akrofrom* as an example, within the 20-year assumed lifetime of the biodigester, the project will deliver useful thermal energy (this is the effective energy used, taking into account stove efficiency) of about 3.5 million kWh at a total cost of US$ 300,000, resulting in a levelised cost of approximately US$ 0.081 per kWh. Delivering the same amount of energy (3.5 million kWh useful energy) with firewood will cost US$ 472,800 over the 20-year period, resulting in a levelised energy cost of approximately US$ 0.135 per kWh. Thus, the levelised cost of firewood is 40% more than biogas, on an energy equivalent basis. The situation is similar for* Asueyi*.

If the gas produced were sold to the community, the NPV over the 20-year lifetime of the project is US$ 78,697 with an IRR of 17.7% in the case of* Asueyi*. The payback is reached in the 8th year. As shown in Table 4, discontinuing the project after 15 years still makes it profitable. Discontinuing in the 10th year, however, results in a negative NPV, rendering the project unprofitable for a commercial enterprise. Also for* Akrofrom* community, the project is profitable for the 20-year and 15-year project duration periods but unprofitable for a 10-year duration. Payback is in the 7th year.

The financial analysis shows that, to the extent that households are willing to purchase the gas for cooking, a larger plant is more profitable than a smaller plant, which agrees with general economic principles. This, however, is dependent on the availability of large quantities of manure in close proximity to the locations where agroprocess wastes are generated. Even though cassava peels are in abundant supply in most cassava processing locations, transporting manure from outside the communities where processing factories are located would increase the project costs.

**Figure 4:**
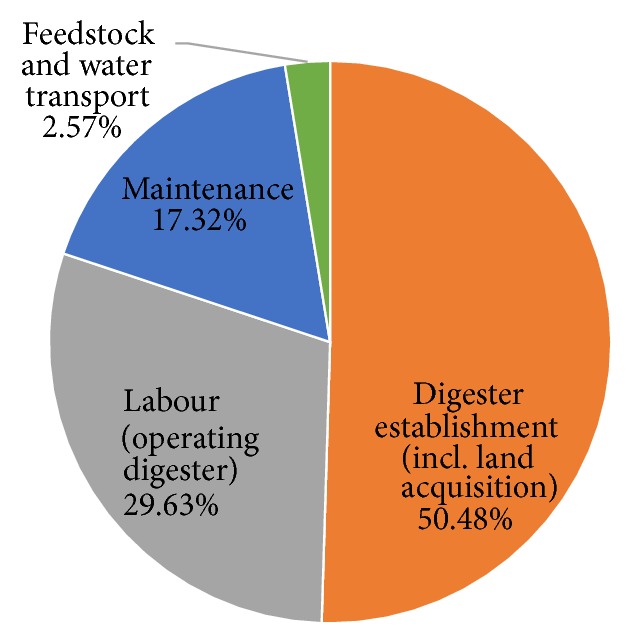
Distribution of total production costs over project lifetime.

The combined production cost for both plants is summarised in Figure 4. Over the lifetime of the project, capital costs constitute 50% of total project costs. This is followed by the cost of labour establishment. Transportation costs are low because feedstock and water are available within the premises of the processing sites which reduces the need for transportation over longer distances. The analysis also assumes manure availability from within the community which avoids the need for higher manure transportation costs.

### 3.3. Job Creation and Income Generation Potential

The important social benefits of a bioenergy programme in an agroindustrial setting are its ability to create employment and therefore provide income for employees engaged to manage and maintain plants. Equally important is the ability of modern bioenergy to displace traditional fuel use in small- and medium-scale agroindustrial settings. Summary of job creation potential and firewood displacement from the two plants are shown in Table 5. It is expected that unskilled jobs will be sourced from within the locality. Details of direct jobs are presented in terms of man-hours per year. The unskilled labour requirement for both projects, in the investment year, is equivalent to 10 people engaged full-time for all business days in the year. In the operating years, the projects would create approximately 4 permanent full-time unskilled jobs and part-time management position for regular monitoring of technical performance. Labour services in the operating years include those for loading of feedstock and monitoring of digester performance and the collection of manure to the project site. The direct unskilled job creation stands at one job per 200 m^3^ digester. This is slightly higher than the calculated direct employment of around one job for 11.7 family sized (ranging between 4 and 15 m^3^) digesters built [25]. The low unskilled job creation is attributable to the fact that feedstocks meant for the digesters are produced on site and would not have to be transported over longer distances.

Income effects are directly related to the number of jobs created on the project. Unskilled labour man-hour rate is estimated at US$ 0.5. For an 8-hour working day, this exceeds Ghana's minimum wage for the year 2014 which is GHC 6 or approximately US$ 2.14 per day (using exchange rate of 1 US$ to GHC 2.81 on May 1, 2014, when new minimum wage was announced) (exchange rate information from http://www.oanda.com/currency/converter/). The hourly wage is also higher than current labour rate in the study communities which is less than US$ 0.3 per hour.

## 4. Discussion 

Wood fuel continues to be the main fuel source in Ghana today, contributing more than 75% to total fuel needs in 2010 [26]. According to data from the Ghana Energy Commission, per-capita consumption of wood fuels in 2013 amounted to 415 kg [27]. Even though per-capita consumption may reduce gradually due to the increasing adoption of gas as cooking fuel, growing population could result in an increase in the national consumption. Presently, it is estimated that cassava processing for* gari* alone contributes about 13% to the total wood fuel consumption. But the production of* gari* is just one way of processing cassava at the agroindustrial level. Other industrial uses, such as the production of starch, are also dependent on the use of wood fuel. Many other small and medium agroindustrial activities such as the production of palm oil and palm kernel oil are very much dependent on firewood as fuel source. The wood fuel needs for these activities would have alarming consequences, looking at the fact that the country's wood resource base is diminishing. Estimates show that Ghana's net increase in forest degradation averaged about 115,000 ha/yr during the period 2000–2005 [28]. To prevent a disaster in the forestry sector, efforts must be made to explore the use of agroprocess residues for energy production. As has been shown in this study, this has the opportunity to not only reduce the amount of wood fuel used in the processing of cassava, but also create job opportunities for poor rural households and add income to these communities. Another important benefit of biogas production is the effluent, which can be returned to cassava and other crop fields as organic fertiliser after appropriate treatment. This extra activity could be considered in order to create a near-zero waste system.

Presently, there is no proper motivation for agroindustries to invest in biodigesters to supplement or replace their processing fuel needs. The state should examine financial structures to assist agroprocessing plants to explore options of deploying biochemical or thermochemical biomass technologies for generating energy from their waste resources. One option is by introducing a funding scheme to provide some capital subsidy. This is one of the tools proposed in the Renewable Energy Law [15] to scale up the uptake of renewable energy in the country. Under the Renewable Energy Law, an RE Fund has been created to provide capital subsidies to renewable energy projects. What government must do now is to ensure the flow of resources into the fund and to provide appropriate funding to projects with bankable proposals. There is also the need for assistance in the preparation of bankable project proposals from agroindustries, to provide them with source funding not only from the RE Fund, but also from bilateral and multilateral donor agencies that offer development assistance to the country.

Apart from subsidies, the state could also use environmental taxes and associated incentives to push for the uptake of bioenergy technologies. The introduction of environmental taxes could encourage companies to shift to cleaner fuels for agroprocessing, especially those that are located within the urban centres whose waste streams have polluting effects on the environment, especially water bodies. Next would be the introduction of a gradual ban on the use of wood fuel for agroindustrial processing, starting from large urban centres. This should go hand in hand with the granting of tax breaks for modern bioenergy interventions. Tax breaks could also come in the form of duty-free clearing of imported bioenergy plants. The Energy Commission, Environmental Protection Agency (EPA), and other appropriate agencies could lump these projects together and trade for carbon credits to partly defray the cost of any subsidies and tax breaks.

This study has shown that even though there could be enough cassava peels for the production of gas, the unavailability of enough manure in cassava processing communities limits the amount of peels that could be utilised. One of the models that could be used to obtain manure for bigger biogas plants is a peel-manure exchange programme where processing plants will come to some arrangement with livestock farmers to convey manure to cassava processing sites in exchange for cassava peels to feed livestock. This could make cheap manure available in large quantities for the production of biogas. In the end, it becomes a win-win situation for both sectors, as livestock farmers have also had difficulty managing their manure [29].

The development of biodigesters to provide modern cooking fuels in rural communities has been a success in Asia with notable success stories in China, India, and Nepal. These success stories were supported by government legislation and were aimed at reducing forest degradation and introducing environmentally friendly fuel to an ever growing rural population. Fortunately, recent legislation in Ghana is supportive of such schemes. To move from the present to the stage envisaged will require substantial funding and it is hoped that government will provide the necessary incentives to make this a reality.

## 5. Conclusions

Agroprocess industries continuously generate waste throughout the year which can be used for the generation of biogas or other energy carriers. This study analysed the possibility of using cassava peels from* gari* production industries for the production of biogas. The study was conducted in two communities in Techiman Municipality in Ghana. The two case study agroprocessing plants in the two communities each process between 7,000 and 8,000 t of cassava per annum, generating an excess of 4,500 t of waste. This study has estimated that a combined total 800 m^3^ digester for both processing plants could displace a little over 300 t of firewood per year and create both skilled and unskilled jobs in the communities. Based on the amount of firewood currently used for* gari* production, it has been shown that, over a 20-year period, utilising firewood will cost 40% more than using biogas, on an energy equivalent basis. In a business-as-usual scenario, this study has shown that approximately 1.3 million tonnes of firewood will be needed by 2030 to produce* gari* in Ghana. The displacement of firewood with gas could have environmental, economic, and social benefits in creating sustainable development.

## Figures and Tables

**Table 1:** Field determined ratio of peels to cassava.

Variety	Peel-to-cassava-root ratio	Moisture content
*Bensere (Yensere)*	0.312	19.9
*Nkruwa*	0.288	20.09
*Dakwari*	0.321	20.22
*Esam*	0.29	19.8
Average	**0.303**	**20.00**
Standard deviation	*0.016*	*0.188*

**Table 2:** Cassava peel and biogas production details.

Parameter	Unit	*Asueyi*	*Akrofrom*
Annual cassava consumption	t	8,000	7,000
Peels generated	t	2,424	2,121
Estimated peels collected for livestock feeding	t	727	1,414
Peels discarded	t	1,697	707
Peels considered for biogas production	t	97	148
Firewood used for *gari* production	w/w	0.85	0.85
Estimated annual biogas production	m^3^	27,463	45,744
Amount of firewood displaced per annum	T	119	198

**Table 3:** Estimates of firewood needed for *gari* production.

Parameter	2015	2020	2025	2030
Projected cassava production (t)	17,149,547	21,066,444	25,877,948	31,788,382
Estimated cassava for *gari* production, 25% of total produced (t)	4,287,387	5,266,611	6,469,487	7,947,096
Estimated *gari* (t)	803,885	987,490	1,213,029	1,490,080
Estimated firewood needed (t)	683,302	839,366	1,031,074	1,266,568

**Table 4:** Key financial variables of the analysis.

Output variable	Project life			Unit
	10 years	15 years	20 years	
*Asueyi*				
NPV	−7,004	35,021	78,697	US$
IRR	8.3	15.0	17.7	%
Digester size	300	300	300	m^3^
Capital cost	90,690	90,600	90,690	US$
Average revenue per year	19,066	25,340	34,259	US$
*Akrofrom*				
NPV	−832	72,550	147,905	US$
IRR	9.9	16.2	18.7	%
Digester size	500	500	500	m^3^
Capital cost	150,791	150,791	150,791	US$
Average revenue per year	31,757	42,207	57,063	US$

**Table 5:** Annual socioeconomic benefits of project.

Socioeconomic indicator	Unit	*Akrofrom*	*Asueyi*
Skilled jobs, investment year	Man-hours	16,088	9,659
Unskilled jobs, investment year	Man-hours	12,873	7,745
Skilled jobs, annual	Man-hours	1,560	1,560
Unskilled jobs, annual	Man-hours	113,843	103,398
Biogas available per year	m^3^	45,744	27,463
Amount of firewood displaced per year	Tonnes	198	119
